# Implementing statistical equating for MRCP(UK) parts 1 and 2

**DOI:** 10.1186/1472-6920-14-204

**Published:** 2014-09-26

**Authors:** IC McManus, Liliana Chis, Ray Fox, Derek Waller, Peter Tang

**Affiliations:** UCL Medical School, University College London, Gower Street, London, WC1E 6BT UK; Research Department of Clinical, Educational and Health Psychology, University College London, Gower Street, London, WC1E 6BT UK; MRCP(UK) Central Office, Royal College of Physicians, 11 St Andrews Place, Regent’s Park, London, NW1 4LE UK; Gartnavel General Hospital, 1055 Great Western Road, Glasgow, G12 OYN UK; Southampton General Hospital, Southampton, SO16 6YD UK; Department of Psychology (26 Bedford Way Building), University College London, Gower Street, London, WC1E, 6BT UK

**Keywords:** Item-response theory, IRT, Statistical equating, Differential item functioning, International medical graduates, Predictive validity

## Abstract

**Background:**

The MRCP(UK) exam, in 2008 and 2010, changed the standard-setting of its Part 1 and Part 2 examinations from a hybrid Angoff/Hofstee method to statistical equating using Item Response Theory, the reference group being UK graduates. The present paper considers the implementation of the change, the question of whether the pass rate increased amongst non-UK candidates, any possible role of Differential Item Functioning (DIF), and changes in examination predictive validity after the change.

**Methods:**

Analysis of data of MRCP(UK) Part 1 exam from 2003 to 2013 and Part 2 exam from 2005 to 2013.

**Results:**

Inspection suggested that Part 1 pass rates were stable after the introduction of statistical equating, but showed greater annual variation probably due to stronger candidates taking the examination earlier. Pass rates seemed to have increased in non-UK graduates after equating was introduced, but was not associated with any changes in DIF after statistical equating. Statistical modelling of the pass rates for non-UK graduates found that pass rates, in both Part 1 and Part 2, were increasing year on year, with the changes probably beginning before the introduction of equating. The predictive validity of Part 1 for Part 2 was higher with statistical equating than with the previous hybrid Angoff/Hofstee method, confirming the utility of IRT-based statistical equating.

**Conclusions:**

Statistical equating was successfully introduced into the MRCP(UK) Part 1 and Part 2 written examinations, resulting in higher predictive validity than the previous Angoff/Hofstee standard setting. Concerns about an artefactual increase in pass rates for non-UK candidates after equating were shown not to be well-founded. Most likely the changes resulted from a genuine increase in candidate ability, albeit for reasons which remain unclear, coupled with a cognitive illusion giving the impression of a step-change immediately after equating began. Statistical equating provides a robust standard-setting method, with a better theoretical foundation than judgemental techniques such as Angoff, and is more straightforward and requires far less examiner time to provide a more valid result. The present study provides a detailed case study of introducing statistical equating, and issues which may need to be considered with its introduction.

## Background

MRCP(UK), the Membership examination of the Royal Colleges of Physicians of the United Kingdom, consists of three separate components, Parts 1 and 2 of which consist of computer-marked multiple choice assessments, and PACES is an OSCE-style assessment of clinical skills, including examination of real patients and communication with simulated patients. This study primarily considers the written examinations, Part 1 and Part 2, for which Part 2 can only be taken after Part 1 has been successfully passed. Standard-setting methods for the written exams have varied over the years, and we will firstly describe those changes, in particular the implementation of a hybrid Angoff-Hofstee method, introduced in 2002 for Part 1 and Part 2, followed by a transition to statistical equating for Part 1 in 2008 and for Part 2 in 2010.

The introduction of statistical equating was generally satisfactory, but several unexpected changes occurred, of which the most striking change was that although the overall pass rates for UK graduates (the reference group) remained stable, the pass rate for non-UK candidates seemed to increase. Any such possible changes need understanding, and therefore in the empirical part of this paper we will analyse data from a series of recent examinations in order to test explanations for the changes which have occurred. The changes can only be properly understood given a background of the methods used before and after statistical equating was introduced, and we will describe them first as a part of the introduction. After that we will describe the various analyses we carried out in looking at the details of the equating process and its possible consequences, in particular considering the issue of differential item functioning in relation to UK and non-UK candidates.

### Overview of standard-setting methods

Many examinations in the past have been norm-referenced, in which a fixed proportion of candidates overall, or of a reference group, passes the assessment. Norm-referenced approaches have been much criticised in the literature [1–5], because they are a ‘relative’ method of standard-setting, which is generally not acceptable for high-stakes examinations since whether or not a particular candidate passes depends not only on their own performance but on the performance of other candidates. Norm-referencing also cannot cope with changes in the standard or quality of candidates, which undoubtedly occurs [6–8]. Instead, absolute methods or judgmental methods of criterion-referencing are preferred, in which a candidate’s performance is judged against a set of explicit performance criteria [9–11]. Methods of setting absolute standards are most often judgemental, being based in the expert judgements of content-matter experts who have read the items and considered how a just-passing candidate (‘minimally competent’, ‘borderline’) should perform on them [9], using methods such as those of Ebel [12] and of Angoff [13]. Although the Angoff method in particular has been much used internationally for the past two decades [14], there are many variants on the method [15], with little consensus about what is important in the way the method is used. More problematic are studies of the details of how examiners make judgements when Angoffing an exam, with strong suggestions that if normative data are provided then these overly influence the judgements, but if they are absent then the judgements are, in effect, close to being random [16, 17]. Indeed, Verheggen et al. suggested that the Angoff method may say more about examiner competence than candidate competence [18]. Some methods of standard-setting are compromise methods, having components of both relative and absolute methods, with the most well-known being the Hofstee approach [19, 20], which takes into account acceptable ranges, however they may be estimated, in the pass mark and the pass rate, and provides a principled compromise between the demands of the two different sets of criteria in a defensible way [21]. A very different approach is that of statistical equating, in which individual items in assessments, particularly those which have been used on two or more occasions, are calibrated against a fixed standard using Item Response Theory [22–25], and those items used to anchor candidate performance in future diets of the exam [26]. Statistical equating is supported by robust statistical and mathematical theory, although of course its suitability depends heavily on the assumptions of the mathematical underpinnings being met in practice. The choice of a particular standard-setting method ultimately is an empirical decision, since, as Kane has emphasised [27], standards which are set have to be valid, in the same sense that the content of the exams on which they are set also has to be validated. Hardly any studies make any serious attempt to validate standards which have been set against empirical data, and particularly not by assessing how different standards result in greater criterion-related predictive validity [27], which is probably the gold standard. In this paper we will compare the hybrid Angoff-Hofstee method which we used previously for stand-setting MRCP(UK), with the statistical equating which replaced it, in terms of predictive validity.

We are also aware that although IRT-based statistical equating is often used for standard-setting examinations, as with NBME and ETS in North America, and by UKCAT in the UK [28], there are very few papers describing the details of its implementation, particularly concerning some of the problems that can arise, and we are aware of no analyses that systematically compare statistical equating with judgmental forms of standard-setting. There are broad statements of approach [29], but little looking back at the details and problems of implementation itself.

### Standard-setting for the MRCP(UK) written examinations

#### Norm-referencing

Standard-setting for Parts 1 and 2 has varied over the years. Historically standard-setting for Part 1 and 2 was norm-referenced [30, 31], although that approach to standard-setting has been criticised in general (see above), and more specifically in relation to UK postgraduate medical examinations and the MRCP(UK) [32, 33].

#### Hybrid Angoff-Hofstee standard-setting

In 2001 and 2002, several major changes were made to the Part 1 and Part 2 examinations. Part 1 was changed from multiple-true-false questions, scored as +1 for correct, -1 for wrong and 0 for not answered, to best-of-five questions, with no negative marking. Part 2 was changed from a mixture of short answers, which had to be marked by hand, and occasional best-of-five items, to an exam consisting entirely of best-of-five items. Standard setting for both Part 1 and Part 2 was also changed to a hybrid Angoff-Hofstee method.

The Part 1 and Part 2 Boards each had their own panels of examiners who carried out Angoff standard-setting (Part 1: n = 5 to 8, median = 7; Part 2: n = 6 to 14; median = 10). The Part 1 standard-setters worked together for all items, whereas the Part 2 standard-setters worked as two teams, the items being divided into two sets, with a number of shared items to allow comparison of standards across the two groups). Each member of a standard-setting group firstly assessed each item on the paper individually, usually in their own time before the main meeting, and they made an estimate of the proportion of just-passing candidates whom it was felt should know the answer to the question. Question papers did not have correct answers indicated, and examiners therefore made these *pre-discussion judgements* without looking at the answers, which were provided on a separate sheet and could be looked at after the judgements were made. At the standard-setting meeting all estimates of the standard for each individual question were displayed to the group and the ‘hawk’ and the ‘dove’ (the examiners who made the highest and lowest estimates of the standard for that question) initiated a general discussion by explaining the reasons for their decisions. When the discussion was complete the examiners made their *post-discussion judgements*, and all examiners were allowed to change their previous estimates. Finally, for questions which had been used in exams before, and hence for which there were normative data, examiners were told the proportion of just-passing candidates who had in fact got the answer correct, and examiners then were allowed to revise their judgements once more to give their *post-normative data judgements*. For new questions the Angoff standard for a question was based entirely on the post-discussion judgements. For questions which had been used before, and where normative data were available, the normative data provided a ‘reality check’, allowing examiners to become aware of how actual performance of candidates on questions could differ from expected performance. For re-used questions in Part 1 the Angoff standard was based on the post-normative data judgments.

Setting of the actual pass mark used a variant of the Hofstee method which incorporated the post-discussion Angoff judgements. A Hofstee method was added to the Angoff method as it was recognised that there is a threat to any assessment if there are large and sudden swings in the pass rate. While such swings may be an indication that the overall performance of candidates might have changed, it is also probable that the real performance of candidates does not typically change so quickly and sharp swings in pass rate might be better interpreted as instability in the standard-setting process itself, rather than in candidate performance. The Hofstee process helps to reduce such swings.

The hybrid method took place in several stages. As in a conventional Hofstee the examiners as a group decided in advance on the acceptable upper and lower limits of the *pass rate*, those estimates being informed by historical data based on norm-referencing. Therefore the pass-rate limits initially for Part One were set at 35% ±5% (i.e. 30% to 40%) and for Part Two were set at 63.75% ±6% (i.e. 57.75% to 69.75%). The Boards could have altered these ranges as time passed, and an original intention had been that they might change, but in practice the range did not change over the time period in which the Angoff-Hofstee method was being used. A conventional Hofstee method also requires that examiners provide an estimate of the acceptable upper and lower *pass marks* for the examination. These estimates in our hybrid method were calculated from the Angoff process. For each examiner an overall Angoff estimate was calculated as the average of all of their judgements for that exam. From the averages for the set of examiners, a standard deviation across the set of individual examiner Angoff estimates was calculated, and a trimmed mean was also calculated, based on the mean examiner Angoff estimates after removing the hawk and the dove examiners. The latter prevented any individual examiner being able to disproportionately influence the standard-setting process by giving particularly high or particularly low standards (although the fact that they contributed to the standard deviation meant that the existence of examiner variability was taken into account). A 95% range was then calculated from the trimmed mean plus and minus two standard deviations, and those values used as the acceptable range of the upper and lower pass marks, and were entered into the Hofstee calculation. Setting of the pass mark then took place in the usual way for a Hofstee procedure, using as a recommended pass mark the point where the inverse cumulative distribution of candidate marks crossed the diagonal line drawn from bottom left to top right of the ‘Hofstee box’. The pass mark thus calculated was only a statistical recommendation to the Board for an appropriate mark, and being only a recommendation the Board could in principle choose to set a different mark, if necessary to take into account other factors, although in practice it never actually did so. When a Hofstee method is used there is always a possibility that the recommended mark is ‘outside of the box’, and in that case it was decided in advance that a pass mark would be used which corresponded to the nearer of the pass rate limits which had been set. In practice this occurred on only 1 out of 16 occasions for Part 1, but for 9 out of 24 occasions for Part 2.

When the MRCP(UK) written exams were revised in 2002 it was intended that the hybrid Angoff-Hofstee method should only be used until sufficient data had accrued to be able to transfer to statistical equating. That process could not take place immediately as the simultaneous transfer to best-of-five items meant that the item bank was small to begin with. The Part 1 and Part 2 exams had three diets per year, and therefore after a number of years there were sufficient items banked to allow statistical equating to be introduced. Statistical equating initially took place for the 2008/3 diet of Part 1 and the 2010/1 diet for Part 2. Prior to equating, the statistical equating process ‘shadowed’ the hybrid Angoff-Hofstee method, and for several diets after statistical equating, a shadow Angoff-Hofstee analysis was also carried out in case there were problems with equating. In practice there were no especial problems and the shadowing was soon discontinued. However re-equating exercises take place every couple of years, with an Angoff exercise on a current diet, followed by comparison with the results of the statistical equating. At that, or indeed any other, point the Boards can choose to alter the overall standard which has been set by statistical equating, although as yet they have not chosen to do so.

#### Statistical equating

Statistical equating utilises the variant of item response theory (IRT) known as Rasch modelling (single-parameter IRT, 1-IRT) in which each item (question) is characterised by a single statistic known as the difficulty [5, 23, 25]. The Rasch model has many conceptual and mathematical advantages over more complex model, and is the standard model used in statistical equating [26]. For any particular examination two separate sets of parameters can be extracted, one set describing the difficulties of the items, and the other set the associated abilities of the candidates. If IRT models for different examinations are fitted separately then the item difficulties and the candidate abilities are standardised separately onto different scales and therefore cannot be compared. However if two or more examinations share items then those shared items can be used to equate the two examinations and to put both the item difficulties and the candidate abilities onto a common scale or metric. In practical terms, two (or more) examinations are analysed using a single IRT analysis, non-shared items being marked as ‘missing’ for those candidates who did not answer them. The result is that all of the items, shared and non-shared, are placed onto the same metric. If any of those items are used in a future examination then their difficulties can be used to anchor the item difficulties for new items and the candidate abilities, making them on the same common scale as the original items which were calibrated [26].

Once IRT is implemented then the pass mark used in a previous examination, the base form, can also be placed onto the common scale, and that same pass mark used in future diets. Without the need for Angoff or other procedures, statistical equating uses information about how candidates performed previously on diets containing shared items, and thereby sets a standard for a new diet, only some items of which have previously been used. The accuracy of statistical equating depends on the number of items in a new examination which are shared with previous examinations. The shared items need not all be from the same previous examination, but can be spread across a range of examinations, which enhances examination security.

The practical procedure for implementing statistical equating for the Part 1 examination, was developed in conjunction with a group of external psychometricians with previous expertise at IRT and statistical equating, and used the specialist IRT software *Winsteps*. While omitting some subtleties and complications, the process can be broadly stated as follows, with Part 1 as an example:

1. The first diet to be statistically equated was 2008/3, with 2008/2 being the *base form*. Since the reference group for MRCP(UK) examinations is UK graduates taking the examination, the estimation of IRT parameters and the statistical equating is restricted to those candidates but the same pass mark can then be applied to all candidates taking the examination.
2. A *concurrent calibration* was carried out for all diets of the examination from 2004/1 up to and including the base diet, which was 2008/2. All items in the bank then had difficulty estimates on the common scale for which candidates had a mean of 500 and a standard deviation of 100, and these item difficulties are placed in the item bank.
3. By comparing those who had passed and failed the base diet the pass mark could be calculated on the new scale as being 521. That pass mark remains for all subsequent diets unless, for whatever reason, the Board decides that it needs to be changed.
4. An *anchored calibration* was then carried out for the first equated diet, which was 2008/3. Selected items which had previously been calibrated were chosen to be used as *anchor items*, to anchor the candidate scores and hence ensure that candidates were on the same common scale as for the base and previous diets. Items which had not previously been used, and hence were not calibrated, could also be calibrated against the anchor items, put on the common scale and added to the bank. Anchor items were chosen for showing appropriate levels of difficulty and item-total correlations, and hence performing well. They should also be representative of the examination as a whole, covering all of the various domains in the blueprint.
5. Candidate scores are calculated for the candidates in the anchored calibration. Since these are on the same scale as in the concurrent calibration, those scoring 521 or more pass the exam, and others fail. That pass mark on the common scale can be converted into a simple percentage of items correct, and that percentage used to score all candidates taking the examination, including those who are not UK first time takers.
6. At the next diet, which was 2009/1 the process repeats, with an anchored calibration based on anchor items which had previously been used, estimation of difficulties for previously unused items and the addition of their difficulties to the bank, calculation of a percentage pass mark based on the equated pass mark of 521, and the calculation of pass or fail for all candidates taking the examination. That process then repeats for each new diet of the examination.

The equating process for Part 2 was similar except that the base form was 2009/3, and the pass mark was set at 425.

#### Different pass rates in UK graduates and non-UK graduates

Soon after statistical equating was introduced there was a suggestion that although the pass rate seemed to have remained constant in UK graduates (the reference group), the pass rate in non-UK graduates had increased, a feature noticed firstly in the Part 1 examination and then also noticed in the Part 2 examination when statistical equating was introduced. It took several years for it to become clear whether the phenomena were real and long-lived, and during that time research was begun to investigate what seemed to be important changes.

## Method

The primary data for the analyses are the results of candidates taking MRCP(UK) Part 1 from 2003/2 to 2013/1, for Part 2 from 2002/2 to 2013/1, and in addition PACES data were available from 2001/1 to 2013/1. Not all data were used for all analyses. Results are generally expressed as pass or fail, with more detailed analyses using individual marks, typically expressed as marks relative to the pass mark. Detailed data on individual items answered correctly were also used for differential item functioning (DIF) analyses. Candidates were divided into those who had qualified at UK medical schools, and who formed the reference group, and other candidates (non-UK). The latter is inevitably a heterogenous group with some candidates being on UK training schemes, some working in the UK and not on training schemes, and many others working in other countries and who have never worked in the UK. Relatively few background data are available for these candidates, beyond age, sex, and date and place of primary medical qualification.

### Statistical methods

Conventional statistical analyses were carried out using SPSS v21, and routine IRT analyses, including statistical equating, were carried out using *Winsteps*. Differential item analyses were carried out using *Bilog-MG v3.0*.

### Ethics

Ethical permission was not required for this study as it involved the routine analysis of educational test data, and hence was exempted from requiring permission under exemption ‘c’ of the UCL Research Ethics Committee (http://ethics.grad.ucl.ac.uk/exemptions.php).

## Results

**Figure 1:**
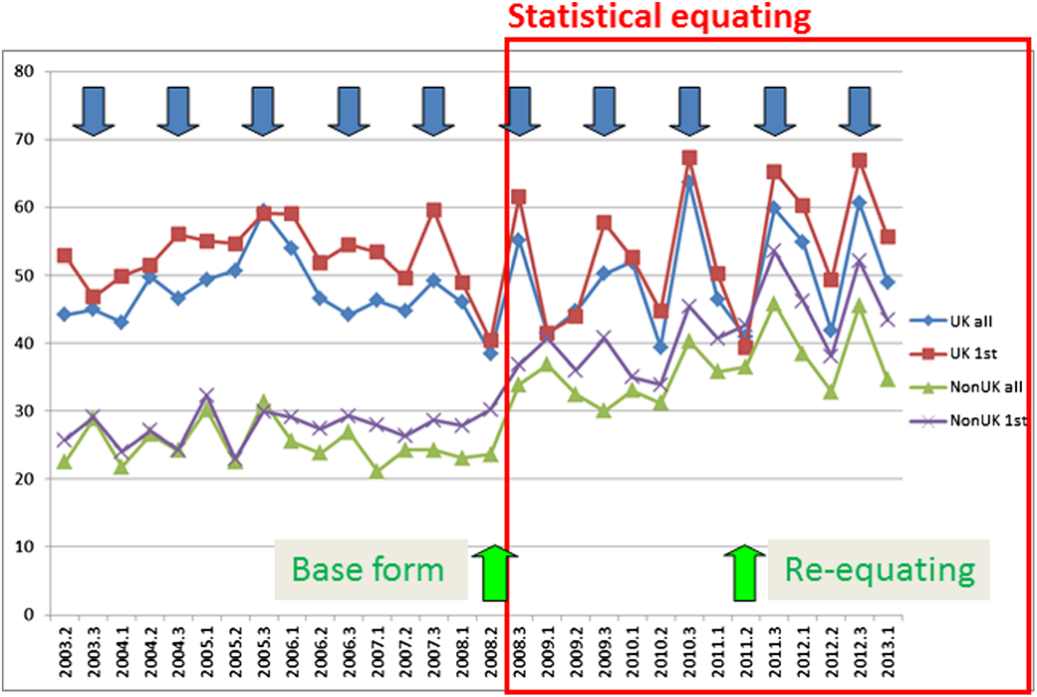
**Pass rates at MRCP(UK) Part 1 in the three diets of each year from 2003 to 2013.** UK graduates and non-UK graduates are shown separately, for all candidates and those at their first attempt. The blue arrows indicate the third diet of each year (see text), and the green arrows indicate the base form and the re-equating exercise. The red box indicates the period during which statistical equating was used.

**Figure 2:**
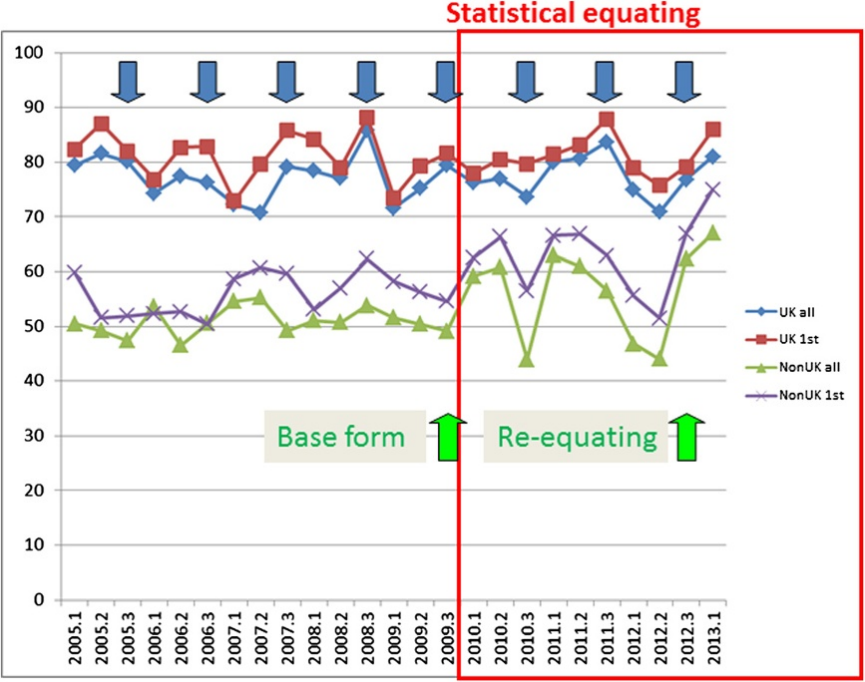
**Pass rates at MRCP(UK) Part 2 in the three diets of each year from 2003 to 2013.** UK graduates and non-UK graduates are shown separately, for all candidates and those at their first attempt. The blue arrows indicate the third diet of each year (see text), and the green arrows indicate the base form and the re-equating exercise. The red box indicates the period during which statistical equating was used.

Figure 1 shows pass rates for MRCP(UK) Part 1 and Figure 2 shows pass rates for MRCP(UK) Part 2. In each figure the pass rates (vertical) are shown for candidates at each diet (horizontal), divided as UK first time takers, all UK candidates, non-UK first time takers, and all non-UK candidates. The red box indicates the diets for which statistical equating was used, and the green vertical arrows indicate the diet used as the base form, and the diet for which re-equating occurred. There are three diets per year and for convenience the vertical blue arrows indicate the third diet of each year. Two separate effects of statistical equating seemed to be apparent.

### Increased pass rate of non-UK candidates

For Part 1 there is a strong suggestion, at least visibly, that although the average pass rate for UK graduates remains stable, the pass rate for non-UK graduates jumps after the introduction of statistical equating and remains higher for the next four years. Similar effects are also apparent for Part 2 but seem not to be as large. Table 1 compares the averaged pass rates for the various groups, pre- and post-equating. Changes for the UK groups are small in all cases. However, for non-UK groups it is clear that the pass rate has gone up by about 1.70× for Part 1, and somewhat less, by about 1.25× for Part 2. A simple Mann–Whitney U-test on the 30 Part 1 pass rates (or the 25 Part 2 pass rates), comparing those before and after statistical equating was introduced, shows a highly significant increase in pass rates for non-UK takers of Part 1, with a significant effect also for Non-UK 1st time takers of Part 2.

**Table 1:** **Average pass rates in UK and non-UK candidates, before and after equating**

		UK all	UK 1st	Non-UK all	Non-UK 1st
Part 1	Pre-equating	47.4%	52.7%	25.0%	27.6%
	Post-equating	50.0%	54.0%	36.2%	41.8%
	Increase post-equating	*1.11x*	*1.06x*	*1.70x*	*1.88x*
	Mann–Whitney U-test	P = .448	P = .647	P < .001	P < .001
Part 2	Pre-equating	77.3%	81.2%	50.9%	56.0%
	Post-equating	77.5%	81.1%	56.5%	63.1%
	Increase post-equating	*1.01x*	*0.99x*	*1.25x*	*1.34x*
	Mann–Whitney U-test	P = .892	P = .723	P = .091	P = .010

Based on 30 pass rates for Part 1 (15 pre- and 15 post-equating) and 25 pass rates for Part 2 (15 pre- and 10 post-equating). Mann–Whitney U tests show exact significance calculated by SPSS.

### Increased annual variation in pass rates

Inspection of Figure 1 also suggests that although the overall pass rate for UK candidates in Part 1 has not altered, there is much more variability after statistical equating has been introduced, with the highest pass rates occurring at the third diet of each year, which is indicated by the solid blue arrows. Table 2 averages results across the various diets in the year, pre- and post-equating, for the Part 1 and Part 2 examinations. Relatively small differences between the 3rd and the 2nd diet of each year pre-equating are exaggerated after equating in the UK candidates, the difference between the 2nd and 3rd diets increasing from 5.1% to 11.7% for UK first time takers after equating. There was much less annual variation in Part 2, and there seems to have been no obvious increase in it after equating. Kruskal-Wallis tests suggest that the annual variations are significant in the UK candidates after equating (but not before), but there is no such effect in non-UK candidates. Without going into details, the annual variation for Part 1 probably results from Part 1 candidates being much more influenced by the annual cycle resulting from the academic year, whereby candidates tend to enter university in October, graduate in June, start working in August, and therefore given the examination regulations, the third diet of the year is the first at which candidates are able to take Part 1, and better candidates probably take the examination earlier. Part 2 takes place later and times for taking the exam are rather more ‘smeared out’ across time and hence the annual variation is less visible.

**Table 2:** **Average pass rates in UK and non-UK candidates by 1st, 2nd and 3rd diet**

		Diet in year	UK all	UK 1st	Non-UK all	Non-UK 1st
Part 1	Pre-equating	1st	47.8	53.3	24.3	28.2
		2nd	45.8	50.2	23.9	26.6
		3rd	48.9	55.2	27.1	28.2
	Post-equating	1st	48.8	52.0	35.7	41.2
		2nd	41.7	44.4	33.2	37.7
		3rd	57.9	63.8	39.1	45.7
	Pre-equating	*3rd – 2nd*	*3.1*	*5.1*	*3.2*	*1.6*
	Kruskal-Wallis test (2 df)		P = .885	P = .217	P = .124	P = .350
	Post-equating	*3rd – 2nd*	*9.2*	*11.7*	*3.4*	*4.5*
	Kruskal-Wallis test (2 df)		P = .009	P = .008	P = .221	P = .146
Part 2	Pre-equating	1st	75.2	78.0	52.3	56.4
		2nd	76.4	81.5	50.4	55.7
		3rd	80.1	84.1	50.0	55.8
	Post-equating	1st	78.0	81.1	59.0	64.9
		2nd	76.2	79.8	55.3	61.5
		3rd	78.0	82.3	54.2	62.1
	Pre-equating	*3rd – 2nd*	*3.7*	*2.6*	*-0.4*	*0.1*
	Kruskal-Wallis test (2 df)		P = .152	P = .160	P = .215	P = .887
	Post-equating	*3rd – 2nd*	*1.8*	*2.5*	*-1.0*	*0.6*
	Kruskal-Wallis test		P = .943	P = .845	P = .546	P = .943

### Explaining the differences in pass rates

Although in Figures 1 and 2 the process over the past four or more years can be seen clearly, that was not a luxury that we had when statistical equating was first introduced and we had but a single post-equating diet. There did appear to be a jump, particularly for the non-UK candidates taking Part 1, but no statistical analysis was possible, and the possibility that it was nothing but a chance fluctuation couldn’t be excluded. That might have been the case for the first few diets, but looking back on the entire series of diets for Part 1 and ten diets of Part 2 there seemed instead, when eye-balling the data, that there was a clear, sudden and sustained increase in the pass rate after statistical equating was introduced, an effect which seemed to be *a step-change.* Inevitably, therefore, the presumption was that this was something to do with the introduction of statistical equating, although quite how was not clear. For other reasons we had also been looking at our written exams in terms of differential item functioning (DIF), particularly when comparing UK and non-UK candidates, and that seemed as if it might offer an explanation for the changes in pass rates, particularly if the question mix of items had changed with the introduction of statistical equating, perhaps due to the need for particular anchor items, or whatever, resulting in more items favouring non-UK rather than UK candidates. DIF was therefore the first place that we looked, particularly given that we anyway wanted to know more about its role in our examinations.

### Differential item functioning

**Figure 3:**
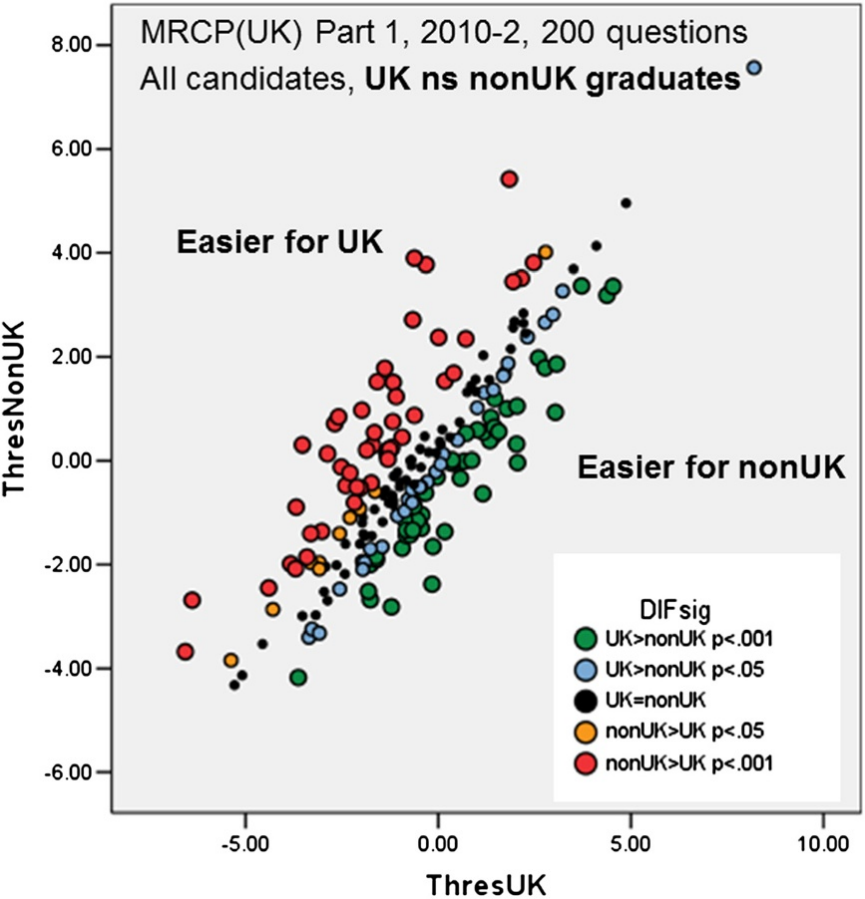
**Example of DIF analysis for a Part 1 diet.** The threshold (difficulty) for each item on the exam is calculated separately for UK candidates (ThresUK, horizontal axis) and non-UK candidates (ThresNonUK), with higher scores indicating more difficult questions. The significance of the difference between the two thresholds is calculated by Bilog and indicated by the colour of the points (see legend).

DIF occurs when, taking overall levels of performance into account, two otherwise equally able groups of candidates perform differently on a particular item or set of items. DIF analyses were therefore carried out for all diets of Part 1 and Part 2, using the program *Bilog-MG*, and diets compared before and after statistical equating. Figure 3 shows the findings of a typical DIF analysis, which is for the 2010/2 diet of Part 1, which had 200 questions. For each item a difficulty parameter is calculated separately for UK and non-UK candidates, and the difficulties are shown as a scattergram, with UK difficulties shown horizontally and non-UK difficulties shown vertically. Significance of differences between UK and non-UK is shown by the size and the colour coding. Many of the items show DIF, but there are approximately equal numbers of items which UK candidates find easier and items which non-UK candidates find easier.

### Distribution of DIF items

**Figure 4:**
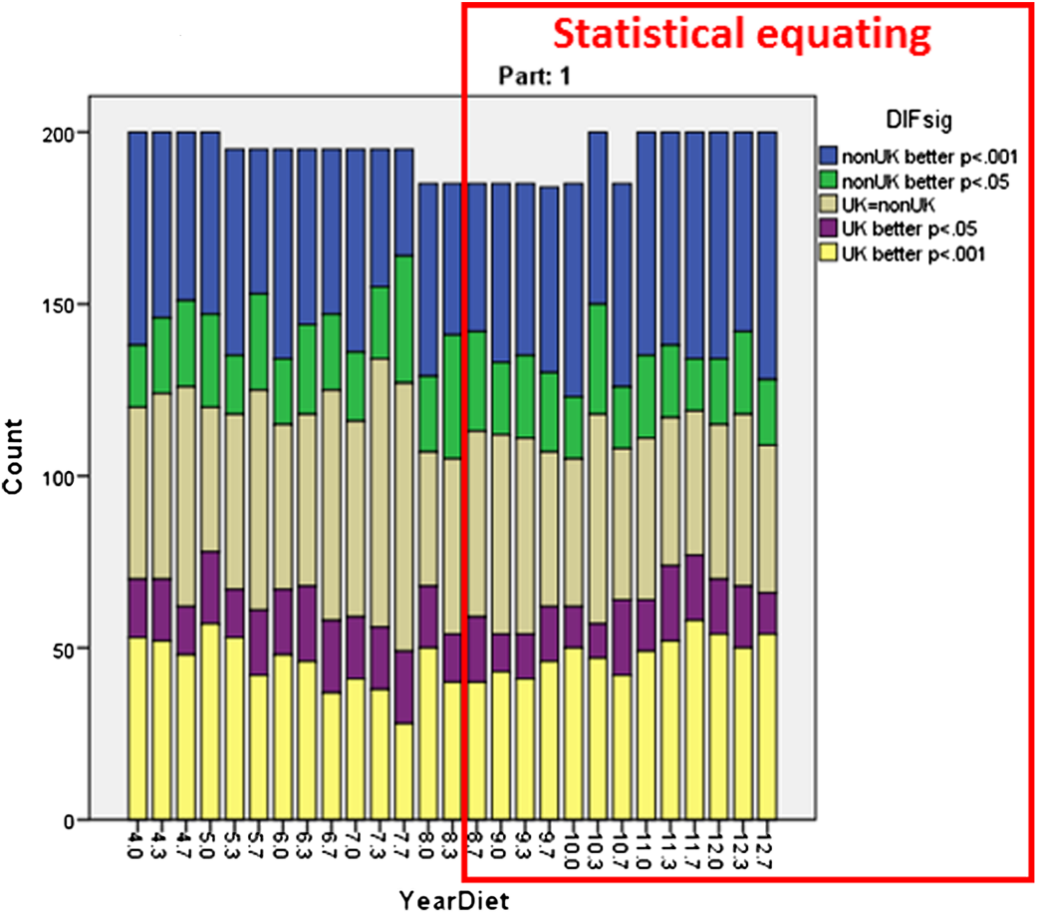
**The numbers of items in each diet of the MRCP(UK) Part 1 exam showing DIF at different levels of significance (see legend).** The red box indicates the period during which Statistical equating was being used. Note that DIF was only calculated for scoring items in the exam, and therefore numbers differ slightly between diets.

**Figure 5:**
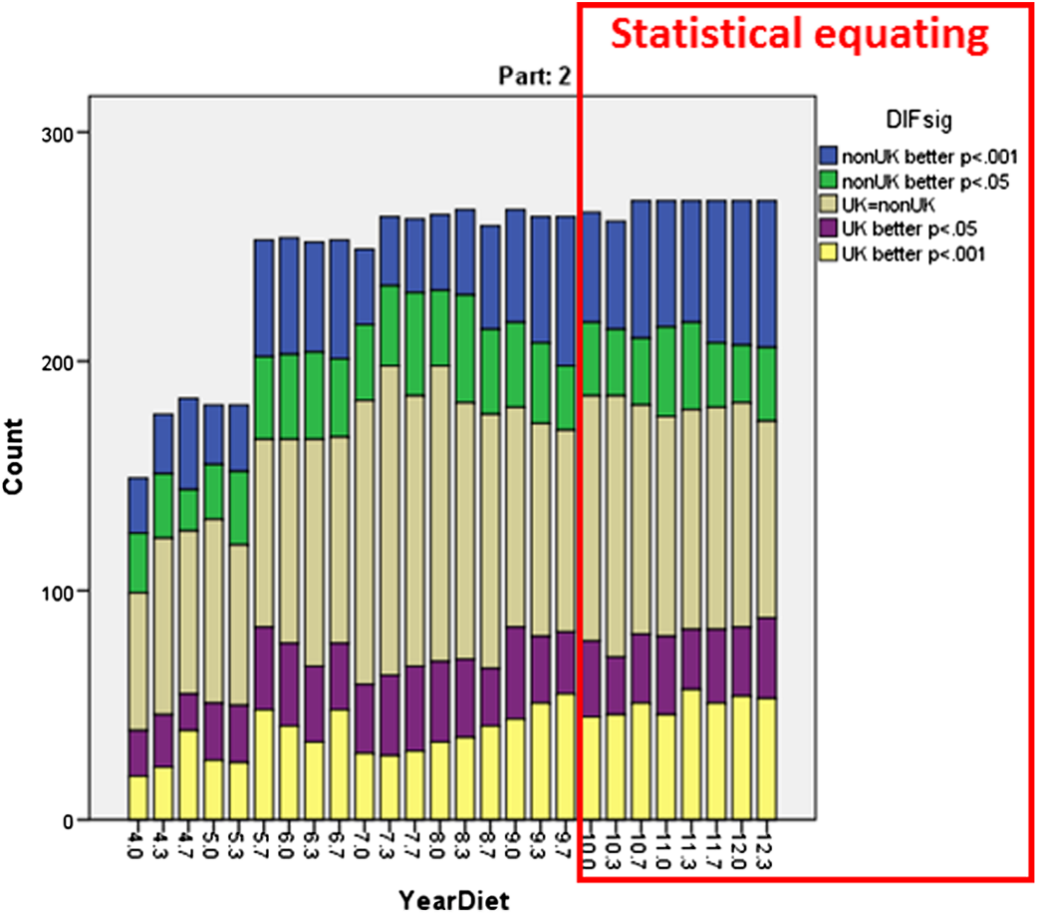
**The numbers of items in each diet of the MRCP(UK) Part 2 exam showing DIF at different levels of significance (see legend).** The red box indicates the period during which Statistical equating was being used. Note that the numbers of items in the Part 2 exam increased in the earlier years, and DIF was only calculated for scoring items in the exam, and therefore numbers differ between diets.

Although in the single diet shown in Figure 3 the items favouring UK and non-UK candidates seem about equally distributed, if a consequence of changing to statistical equating was that the proportion of items favouring non-UK candidates had also changed, then the pass rate of non-UK candidates could increase while the pass rate of UK candidates remained stable. Figures 4 and 5 show the overall proportions of items in a range of Part 1 and Part 2 diets. For Part 1, 52% of a total of 5329 items showed DIF with p < .001, UK candidates performing better on 24.0% of items and non-UK candidates on 28.0% of items. A similar pattern was found for 6385 items in Part 2, 34.9% of items showing DIF with p < .001, 18.4% of items with non-UK candidates performing better and 16.5% of items with UK candidates performing better. Particularly clear in Figures 4 and 5 is that there is no obvious change in the pattern of DIF before and after statistical equating was introduced. A formal test can show that, there being no difference in DIF for items used before statistical equating was introduced and after (Part 1: t(5224) = .054, p = .957; Part 2: t(6383) = 0.001, p = .999). A change in the distribution of DIF cannot therefore be the explanation for the changes in pass rates.

### Anchor items

**Figure 6:**
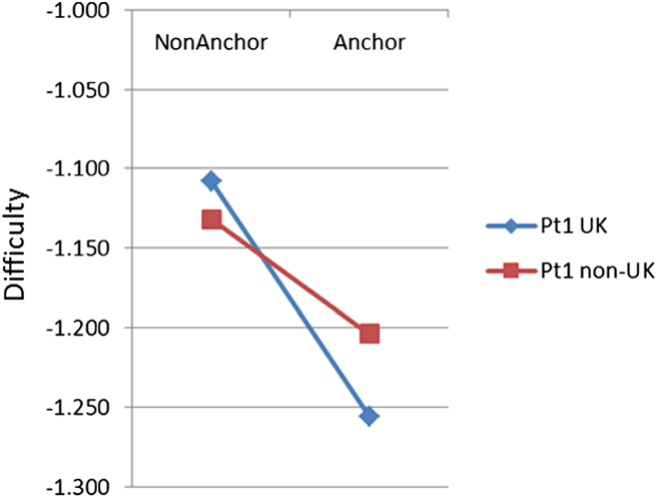
**Mean threshold scores for anchor and non-anchor items by UK and non-UK candidates for the MRCP(UK) Part 1 exam.** See text for further details.

**Figure 7:**
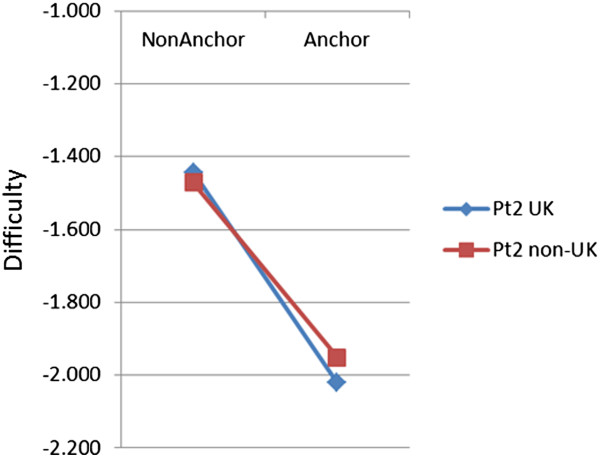
**Mean threshold scores for anchor and non-anchor items by UK and non-UK candidates for the MRCP(UK) Part 2 exam.** See text for further details.

Anchor items are important in statistical equating, as they are used to equate performance across different diets of the exam, and they themselves are usually chosen in terms of good conventional item statistics (high item-total correlation and an appropriate difficulty level). On average 33.2% of items in Part 1 and 29.6% in Part 2 were anchor items. Anchor items are also chosen to be representative of the examination as a whole, and therefore their DIF should also be representative of the examination, and there can potentially be problems in equating if anchor items are not representative. We therefore carried out a systematic examination of the difficulties of anchor and non-anchor items. Figures 6 and 7 show the difficulties of anchor and non-anchor items for UK and non-UK candidates in Parts 1 and 2 of the exam. For both Part 1 and Part 2 a cross-over can be seen, with anchor items being easier (a lower difficulty) for UK candidates but non-anchor items being easier for non-UK candidates. A formal statistical analysis used a repeated measures analysis of variance to examine the difficulty of 7450 items, classified by Part (P; 5041 Part 1, 2409 Part 2), by being before or after equating (E), and by whether they were or were not anchors (A), all of which are between item measures, and by UK vs non-UK candidates (UK), which was a within-item variable. Degrees of freedom for all F values are 1 and 7742. Not all effects and interactions are of substantive interest for considering Figures 6 and 7. Anchor items were easier than non-anchor items (A: F = 18.6, p < .001), Part 2 items were easier than Part 1 items (P: F = 19.2, p < .001), and the PxA interaction was also significant (F = 11.5, p < .001), the difference between anchor and non-anchor items being smaller in Part 1 than in Part 2. UK candidates found items easier than non-UK (UK: F = 880.4, p < .001), the effect differing between the Parts (UKxP: F = 14.6, p < .001). For present purposes the most important test was of the interaction of being an anchor item and candidate type, and that interaction was highly significant (UKxA: F = 16.34, p < .001), the effect being somewhat different in size for Part 1 and Part 2 (UK × A × P: F = 7.2, p = .008). Post hoc tests confirmed that the UK × A interaction was significant in both Part 1 (F(1,5037) = 4.28, p = .039) and Part 2 (F(1,2405) = 8.42, p = .004). Few of the remaining effects are of interest, but we note that the E, P×E, A×E, and UK×P×E effects were all non-significant, and there were significant effects for P×A×E (p = .021), UK×E (p < .001), UK×A×E (p = .002) and UK×P×A×E (p = .002). Certainly it seems that anchor items are behaving differently in the UK and the non-UK candidates.

The different behaviours of anchor and non-anchor items are interesting, and some of the implications will be left until the discussion. For the present, though, it is not clear that that difference alone can explain the apparent difference in UK and non-UK pass rates before and after equating. The argument is simple. Once a diet has been sat then each candidate receives a mark, which can be construed as a simple percentage of items correct, one of the important features of 1-IRT being that there is a monotonic mapping of candidate ability scores onto percentage of items correct. Statistical equating sets a pass mark using data from the UK candidates, and that can be converted into a percentage mark, which is entirely equivalent. All candidates at or above that mark pass the exam and all candidates below it fail, irrespective of whether they are UK or non-UK. Even if the pass mark is not set correctly, for whatever reasons, perhaps due to artefacts involving anchor items, that cannot influence the relative distribution of UK and non-UK candidates at particular levels who pass or fail the exam. If a UK and a non-UK candidate score 65% then either both must pass or both must fail. The only conclusion, therefore, has to be that *if there is apparently an increase in the pass rate of non-UK candidates after statistical equating was introduced, then that can only be because the non-UK candidates have got better*. At that point, we have to go back to the original premise and ask what are the reasons for believing that the changes in non-UK pass rates occurred at the same time as statistical equating was introduced.

### The timing of the increase in pass rate of non-UK candidates

**Figure 8:**
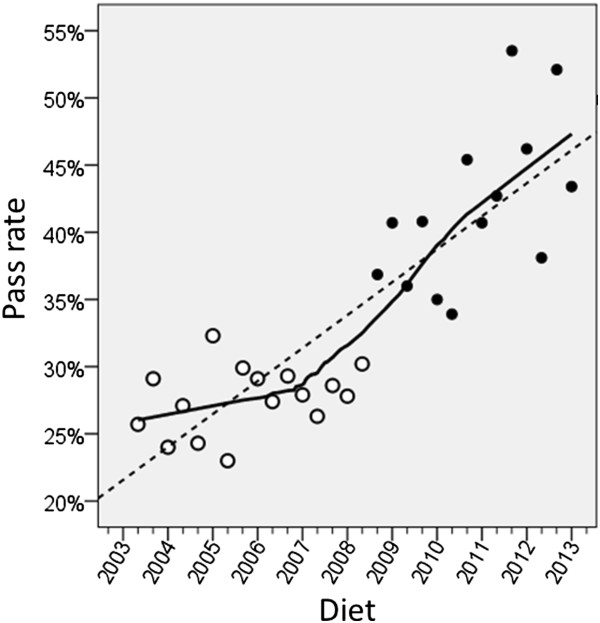
**Pass rate for non-UK first-time takers of MRCP(UK) Part 1 plotted against diet.** “2007” indicates the 2007/1 diet, with other minor tick marks indicating the second and third diets of the year. Open points are pre-statistical equating, and solid points post-statistical equating. The dashed line is a conventional linear regression, and the solid line is a loess curve.

In Figure 1 it seems clear that the pass rate of the non-UK candidates increased after statistical equating was introduced, and it seems straightforward to describe that as a ‘step-change’. However the fallacy of *post hoc ergo propter hoc*, that because A is followed by B then A must have caused B, has been recognised since classical times. At this point it is necessary re-examine the data of Figures 1 and 2 more carefully, to assess the evidence for a step change. A step change is precisely that – the values are around one mean and then after the change they are around a second mean, the only change being at the transition when it is immediate. A more careful examination of the data suggests that in fact what is seen is not a step change. In particular, if one looks at the non-UK pass rate post-equating there is a significant correlation of the pass rate with the date of taking (Part 1 Non-UK 1st attempt, r = .589, n = 14, p = .027). The implication is that the data may not be showing a step-change but a continuing increase in pass rate. To explore that further, Figure 8 shows the same data from Figure 1 for non-UK first time candidates, but replotted. The numbers are all identical but the picture now looks very different, in large part because the introduction of statistical equating has not been emphasised and the lines are fitted to all of the data. A line has been fitted through the entire data set, either using linear regression (the dashed line), or an exploratory, loess curve (the solid line). Post-equating points are indicated by solid circles. Overall the impression is of a pass rate that is continually increasing with time across the whole data range, with perhaps some steepening of the curve, but that steepening seems to begin at about the 2007/1 diet, according to the loess curve. That was tested more formally by firstly fitting a single regression line to the data, the slope of which was highly significant, with a slope of 2.45 percentage points per year (p < .001). A model was then fitted using the non-linear regression program in SPSS, with a constant A, a slope B for diets up until a date P, and a slope C for diets after date P. The model was an almost significant improvement on a single line (F(2,26) = 3.51, p = .089), but the slope B was not significantly different from zero. A reduced model with a slope of zero before the date P, and a slope of C after P was significantly better than a simple straight line (F(1,27) = 6.977, p = .013). For that model the slope C was highly significant (3.55% per year (p < .001). The estimate of P, the point of the break or the dog-leg in the curve, is 2006.95, equivalent to the 2007/1 diet. The confidence interval of P was 2005.7 to 2008.2, equivalent to diets 2005/3 to 2008/2. It seems that non-UK candidates have begun to behave differently, but that was not from 2008/3 onwards, when statistical equating was introduced, but earlier than that.

**Figure 9:**
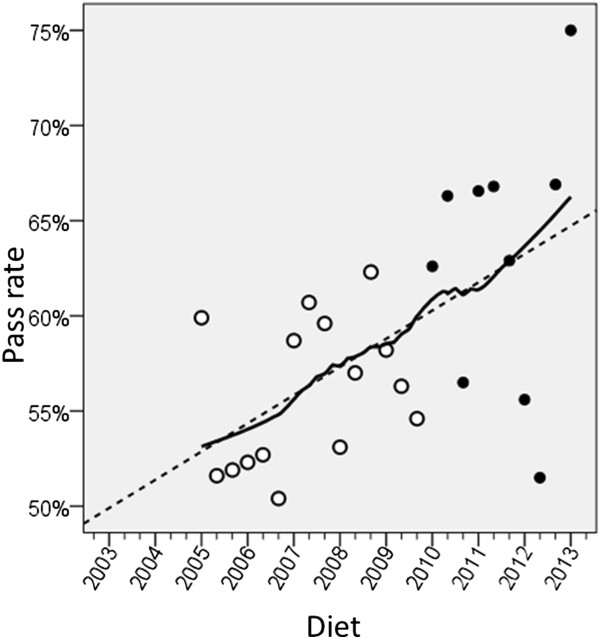
**Pass rate for non-UK first-time takers of MRCP(UK) Part 2 plotted against diet.** “2007” indicates the 2007/1 diet, with other minor tick marks indicating the second and third diets of the year. Open points are pre-statistical equating, and solid points post-statistical equating. The dashed line is a conventional linear regression, and the solid line is a loess curve.

Figure 9 shows an equivalent plot to Figure 8 but for Part 2 non-UK takers on their first attempt. The linear regression which is dashed suggests that the pass rate had been rising across the entire time period, and the loess curve is very similar indeed. Regression of the pass rate on diet showed a very significant linear trend (t = 3.435, 23 df, p = .002), the rate rising by 1.48 percentage points per annum. Adding in a constant to indicate statistical equating did not significantly improve the model (p = .414), and neither did allowing the slope to differ before and after equating (p = .958). Finally, fitting a dog-leg, as was carried out for Part 1, did not improve the fit over a single regression line. The conclusion has to be that the pass rate for Part 2 was increasing before statistical equating was introduced, and it continued to do so afterwards.

Despite the fact that originally it had seemed there was a step-change in Part 1 and Part 2 after statistical equating was introduced, a more careful re-scrutiny of the data over a longer time period than the first few diets suggests that there was not a step-change, but rather, for reasons that are not clear, from about 2007/1 onwards the non-UK candidates began systematically each year to have a higher pass rate in the Part 1 exam. In addition the pass rates in Part 2 had been rising over a longer time period. At it happened, the pass rates for the diets immediately before equating, for both Part 1 and Part, happened to be low, and those immediately after happened to be relatively high, but those can be seen as nothing more than random fluctuations. However, while those changes did produce the strong visual impression of a step change, particularly when data were only available for one or two diets after equating was introduced, a rather different interpretation is required for the data over the longer time period.

### Predictive validity of Parts 1 and 2 before and after equating

Statistical equating was introduced because there were strong theoretical reasons for believing that it was a more robust, more justifiable method of standard-setting than were the subjective judgements used by the Angoff method. If that is truly the case then marks obtained by candidates after equating should have better predictive validity than marks obtained before equating. If a standard-setting method is less valid then a higher proportion of candidates should pass due to chance or because the standard is set at the wrong level than is the case for a more valid method, the passing candidate marks in effect containing different amounts of measurement error. On this argument it should be noted that the lower predictive validity occurs across diets, rather than within them, and that there will be no expected change in reliability within diets as all candidates, passing and failing, contribute to the estimate of reliability.

Table 3 shows correlations between performance on Part 1 and Part 2 and also with performance on PACES, the clinical assessment of MRCP(UK). In all cases the analyses consider the first attempt at an assessment, first attempt being the best predictor of future outcomes [34]. The most important predictive validity concerns Part 1 predicting Part 2, since both assessments are knowledge assessments and hence should be strongly related. Before statistical equating, there was a correlation of .605 between Part 1 and Part 2 results, but for candidates taking both parts after statistical equating was introduced the correlation was .623, the difference being statistically significant, as is also the case for just UK and just non-UK candidates (and indeed those differences are more significant than for all candidates).

**Table 3:** **Correlations of Part 1, Part 2 and PACES results, before and after equating of Parts 1 and 2**

		Part 1 not equated and Part 2 not equated			Part 1 equated and Part 2 equated			
		r	SE	N	r	SE	N	Significance
Part 1 predicting Part 2	**UK**	**0.618**	**0.008**	**6347**	**0.651**	**0.009**	**3865**	**P = .007**
	**non-UK**	**0.553**	**0.008**	**7361**	**0.614**	**0.010**	**4150**	**P < .001**
	***All***	***0.605***	***0.005***	***13708***	***0.623***	***0.007***	***8015***	***P = .040***
Part 1 predicting PACES	UK	0.305	0.010	7875	0.320	0.019	2172	P = .493
	non-UK	0.276	0.010	8648	0.283	0.027	1183	P = .807
	***All***	***0.385***	***0.007***	***16523***	***0.311***	***0.016***	***3355***	***P < .001***
Part 2 predicting PACES	UK	0.285	0.012	5957	0.270	0.020	2180	P = .516
	non-UK	0.233	0.012	5822	0.202	0.028	1195	P = .306
	***All***	***0.309***	***0.008***	***11779***	***0.211***	***0.016***	***3375***	***P < .001***

Pearson correlations (r) are shown separately for UK candidates, non-UK candidates and All Candidates (shown in italics), for candidates where both Part 1 and Part 2 were passed before statistical equating was introduced and for candidates for whom both Part 1 and Part 2 were statistically equated.

The significance of the difference in correlations is computed using a standard test [35], and rows where before and after correlations are significant are shown in bold.

For completeness, Table 3 also shows correlations of Part 1 and Part 2, pre- and post-equating, with PACES, which is the clinical examination of MRCP(UK). Correlations are rather lower than between Part 1 and Part 2, being in the range .20 to .39, compared with .55 to .65 for Parts 1 and 2, and that is to be expected since Parts 1 and 2 are knowledge assessments, whereas PACES is an assessment of clinical skills. The pattern of correlations is also very different. Considering all candidates, the correlations have gone down (.385 to .311 for Part 1 predicting PACES, and .309 to .211 for Part 2 predicting PACES). It could be argued that might be expected if Parts 1 and 2 are now more valid for knowledge-assessment. However, a more detailed scrutiny of Table 3, comparing UK and non-UK candidates finds that none of the four groups (UK or non-UK on Part 1 or Part 2 for predicting PACES) shows a significant change in correlation, with two cases showing increases and two showing decreases after statistical equating was introduced. Graphical exploration showed the discrepancies between the UK, non-UK and All correlations reflected differences in the overall means of UK and of non-UK on the Part 1, Part 2 and PACES measures, and hence the ‘All’ correlations contained two separate components, one reflecting within group correlation and the other reflecting between group differences in means. Considered overall, the UK and the non-UK correlations are probably the most valid, and based on them then the conclusion has to be that there is no change in predictive validity of PACES after statistical equating. Statistical equating has however improved the predictive validity of one knowledge test upon another, from Part 1 to Part 2, and that increased validity is equivalent for UK and non-UK candidates.

### Are repeated questions easier on a second or third usage because of item leakage?

There are many threats to the validity of examinations, and one that must always be considered is leakage of an item bank that is meant to be confidential. When items are used then candidates can, and sometimes do, try to remember the items, for the later use of themselves and others. If items are systematically leaking then the prediction is that they will be easier on a second or later occasion than on their first use. That hypothesis can be tested using IRT. We systematically looked at all non-anchor items used on two or more occasions after the introduction of statistical equating. Table 4 shows statistics on those items, with first, second and third usage for Part 1, but only first and second usage for Part 2 (there being no items used three times in Part 2). The correlation of difficulties across the two occasions is good (mean = .704; range = .604 to .755) suggesting that item difficulties are stable across time. The difference in difficulty across occasions is small (mean = -.034, range = -.143 to .058), and none of the differences reach statistical significance. There is therefore no evidence that items become easier across repeated uses, suggesting that leakage of items is unlikely to be explaining the changes across time shown in Figures 8 and 9.

**Table 4:** **Difficulty of repeated non-anchor items**

	***Occasion***	***Mean difficulty***	***SD difficulty***	***Occasion***	***Mean difficulty***	***SD difficulty***	***N***	***Correlation***	***Difficulty difference***	***t***	***df***	***p***
*Part 1*	*First*	-.035	.891	*Second*	-.045	.941	477	.742	.001	.318	476	.751
*Part 1*	*First*	-.210	.859	*Third*	-.157	.826	55	.755	-.053	-.664	54	.510
*Part 1*	*Second*	.000	.905	*Third*	.143	.651	12	.714	-.143	-.779	11	.452
*Part 2*	*First*	-.065	.891	*Second*	-.123	1.001	279	.604	.058	1.076	278	.283

Difficulties (1-IRT) of non-anchor items used on two occasions (first and second, first and third, or second and third usage), shown as mean and SD on each occasion, with mean difficulty, and paired t-test for difference in difficulties across occasions.

## Discussion

Statistical equating was introduced for the MRCP(UK) written examinations during 2008 and 2010. This change was motivated in large part by a long-term appreciation of the theoretical advantages of statistical equating for a large-scale multiple-choice examination taken by many candidates where adequate data would be available to make robust the complex calculations used in IRT. Norm-referencing, as had been used for MRCP(UK) Parts 1 and 2 until 2002, is vulnerable to shifting standards outside the control of the exam board [6, 7], and the major changes of 2001/2 had the intention to introduce statistical equating to avoid such problems. Statistical equating could not however be introduced immediately as the format of exam questions had also changed, meaning that no large-scale database existed on which IRT could be carried out. The Angoff-Hofstee judgemental method was therefore used as a provisional standard-setting method until, by 2008, a sufficient number of questions were in the database to allow the use of statistical equating, firstly in the larger Part 1 examination, and then two years later in the somewhat smaller Part 2 examination. A benefit of the change to statistical equating was that the Angoff method is expensive in terms of examiner time, a typical diet requiring a whole working day, and perhaps longer, on the part of from seven to ten examiners. In addition examiners often did not feel that their judgements, despite being statistically reliable, were robust, the suggestion often being made that numbers are “being plucked from thin air”. Statistical equating also takes time, but on the part of one or two skilled psychometric staff, rather than five-times as many active clinicians, and it has a more robust theoretical underpinning than the Angoff method.

For UK graduates, the group who formed the reference category, the transition to statistical equating was smooth for both Part 1 and Part 2, and the overall annual pass rates remained stable, as can be seen in Figures 1 and 2. The pass marks set during the concurrent equatings have been kept at the same level (521 for Part 1 and 425 for Part 2) for the years since equating was introduced, with the standard being reviewed and re-equated once for both Part 1 and Part 2. The standard-setters and the Boards of course have the option, after reviewing a wide range of evidence, including statistical data, the opinions of candidates, trainers and employers, and an Angoff recalibration, of deciding that a change in the pass mark is desirable. That is a legitimate function for an examination board to carry out, since pass marks ultimately are not set by computer programs or by statisticians but by the considered judgements of well-informed Boards of Examiners. For the majority of the time, particularly outside of the review process, the statistical equating process runs smoothly, without the need for extensive intervention for the Board, and provides reasonable and justifiable pass mark recommendations which the Board finds acceptable. Statistical equating did however result in two outcomes which were unexpected, and also provided the opportunity to assess whether there was a greater validity of the examination process post-statistical equating.

### Increased annual variation in pass rates

The less difficult change to explain was the greater swings in the pass rate within the annual cycle for UK Part 1 candidates, which can be seen clearly in Figure 1. This greater variation arises in part because in the absence of a Hofstee box there are no limits on the pass rates than can result from equating. However on only one occasion for Part 1 was the Hofstee recommended mark ‘outside the box’, and that does not therefore seem to be the explanation. The most reasonable explanation for the difference between diets in Part 1 is that it reflects a genuine difference between the different groups first taking the exam in the three diets of each year, a difference which would be largest in UK first-time takers (and the effect can, to some extent, be found in the pre-equating results in Table 2), but which statistical equating makes much clearer. A genuine difference in performance was therefore either being ‘covered up’ by the judgement-based standard-setting, or else it was not being found due to noise in the process. Implicit in that analysis is a criticism of the Angoff process itself, and the Angoff process will be returned to later.

### Validity pre- and post-statistical equating

The use of statistical equating, like any procedure for running an examination, is only justifiable in so far as it results in a more valid outcome. The analyses of the predictive validities of Part 1 and Part 2 before and after statistical equating was introduced (Table 3), show that statistically equated Part 1 is a significantly better predictor of statistically equated Part 2 than was Angoff-Hofstee’d Part 1 a predictor for Angoff-Hofstee’d Part 2. That is a strong indicator that statistical equating is better than judgemental equating using procedures such as Angoff and Hofstee. If statistical equating is better than judgemental equating, as MRCP(UK) used previously, then the conclusion once more has to be that the greater annual variation in Part 1 is real, that the quality of the candidates is indeed better at diet 3 than the other two diets, and that using a judgemental standard-setting method had failed to find that effect reliably.

### The change in the pass rate of non-UK candidates

For the exam boards, the biggest surprise in introducing statistical equating was the apparent sudden increase in the pass rate amongst non-UK candidates. The change was not anticipated, and there was no immediate explanation for the change, which visually appeared very clear. Only now, five years after the introduction of statistical equating to Part 1, and three years after introducing equating to Part 2, is a proper explanation possible, in large part because it needed that longer time period to collect an adequate series of data for testing explanations. The explanation seems simple. There is no need to explain the sudden step-change in Part 1 or Part 2 pass rates because there was no sudden step-change in pass rates. For the two diets of 2008/3 and 2009/1 it did look as though non-UK pass rates were increasing, but that was a very small sample on which to work. A more detailed analysis over nearly five years suggests that pass rates for Part 1 non-UK candidates had started to rise before statistical equating and had continued to rise each year after equating was introduced, and had been rising for a longer period for Part 2. None of that is compatible with any straight-forward artefact arising from equating itself. In all probability, all of those involved, including ourselves, were subject to a cognitive illusion, interpreting changes in Part 1 from 2008/3 as being caused by changes in the method of equating because we knew that the method of equating had changed. Standing back and taking a longer view shows a different picture. In that different picture the pass rate of non-UK candidates at Part 1 has not only increased from 2007 to 2013, but because statistical equating has been used from 2008/3 onwards, it has to be concluded that the improvements are real. Similarly, statistical equating had not altered what was actually a longer term increase in the pass rate for Part 2. There is no theoretical reason why statistical equating alone could produce such an effect, there is no evidence that it has done, and since statistical equating produces better predictive validity then the improved performance at both exams has to be taken as genuine. The reasons for it are another matter, but the non-UK candidates are a heterogeneous group, some being UK trainees, some never having worked in the UK, and non-UK candidates are taking the MRCP(UK) exams for a host of reasons. In addition medicine within and outside the UK over the same time period has inevitably changed in various ways due to a range of pressures. There is no obvious reason why the performance of non-UK candidates should have stayed constant, albeit the cause for changes is as yet not understood. What the changes are not due to is an artefact resulting from the introduction of statistical equating, and indeed, it is in part the statistical and theoretical robustness of statistical equating which means that the increase in part rates can be accepted as real.

### Differential item functioning

The existence of DIF in Part 1 and Part 2 has been known about for many years. Differential Item Functioning is generally seen as an aspect of test performance which is ‘construct-irrelevant’ and hence a threat to the validity of the test [36, 37]. If DIF items are relatively rare in a test, then it is commonplace to remove them from the assessment in order to ensure test fairness. Figure 3 however suggests that UK-nonUK DIF is far from rare, and the systematic survey of a large number of diets found that about 52% of Part 1 questions and about 35% of Part 2 questions showed DIF with p < .001. In that situation it is not straightforward simply to delete all questions which show DIF, not least, because as Chu and Kamata have said, “deleting DIF items from the test could deteriorate construct validity”(p.343) [38].

Interpreting DIF crucially depends on what is seen as construct-relevant or construct-irrelevant. Consider a group of UK doctors who as a part of their training have worked in different hospitals on units with differing specialities, and some have worked on wards specialising in respiratory medicine and others have not. Those two groups would probably differ in their performance on exam questions concerned with respiratory medicine. That is differential item functioning, but it is not construct-irrelevant variation if the examination blueprint includes knowledge of respiratory medicine, and hence those respiratory medicine items cannot be omitted. Scrutiny of the content of Part 1 and Part 2 items which DIF between UK and non-UK candidates suggests, as a crude approximation, that questions favouring UK candidates tend more to be on high-tech laboratory-based aspects of medicine, whereas questions favouring non-UK candidates are more typically on the clinical presentation, diagnosis and treatment of conditions which once were common in the west, but are now much rarer. Similar differences have been reported by the NBME [39], finding that, compared with US/Canadian candidates, IMGs at Step 1 scored better in anatomy, embryology and pathology and worse at behavioural science and genetics, and at Step 2 CK IMGs performed better at surgery and worse at psychiatry (pp.42-3), those differences probably reflecting differences in training and experience. Amongst postgraduate examinations, MRCP(UK) is not unique in finding DIF between home graduates and IMGs, Dugosh et al. [40] reporting that in the Geriatric Certifying Examination of the ABIM (American Board of Internal Medicine), “8% - 13% of items … were flagged for intermediate to large DIF [no definition provided]”, with about half favouring US medical graduates and the other half favouring IMGs.

DIF clearly has to be seen as a fact of life for medical examinations, and standard-setting ought, in principle, to take account of it, although as Chu and Kamata point out, DIF and standard-setting are two mostly entirely separate areas of research and analysis in the literature with little overlap between the two.

Although DIF undoubtedly occurs in Parts 1 and 2 of MRCP(UK), the extent of it did not seem to change after statistical equating was introduced (Figures 4 and 5), and nor did the balance of questions favouring UK or non-UK candidates. If the extent or nature of DIF had changed as a result of statistical equating then it might have resulted in changes in pass rate amongst non-UK candidates. The extent of DIF didn’t change though, and hence DIF is not relevant to understanding why the pass rate of non-UK candidates has increased in recent years.

### DIF, anchor items and standard-setting

Although there are some, very technical, approaches to standard-setting in the presence of DIF [38], most accounts of statistical equating mention it little. The statistical equating used in Part 1 and Part 2 is what Kolen [26] describes as a, “non-equivalent groups design”, groups at different diets being allowed to differ in overall ability, with differences being estimated by means of marker, anchor or common items. Kolen emphasises in particular, that, “for this design to work well, the common items need to represent the content and statistical characteristics of the total test” (p.20). Kolen also provides a worked example showing how equating in principle can go wrong when common items are not representative of items in general. The analyses shown in our Figures 6 and 7 do show that the anchor items which were used were not entirely representative of the items on the paper as a whole, anchor items being easier than non-anchor items overall, with anchor items also being somewhat harder and non-anchor items being somewhat easier for non-UK candidates. Anchor items are also chosen, with good justification, as having higher point-biserial correlations (and it would make little sense to use poorly correlating items as anchors). The impact of all of those differences on statistical equating is not clear at present. Once again, though, and however interesting and important it may be, it could not be responsible for any simple step-change in pass rates after equating was introduced, and neither can it explain the continuing increase in pass rates of non-UK candidates.

### How should the apparent change in pass rates for non-UK candidates have been responded to?

It would have been easy in the diets after statistical equating was introduced to Part 1 to have jumped to the conclusion that equating was artefactually, in some way unknown, resulting in the apparent pass rate increase. When the effect subsequently also seemed to have been there for Part 2 then the argument for making changes to statistical equating might have been stronger still. Making changes would not have been justified however, partly on the grounds that there was insufficient evidence to be confident of the changes which were occurring, and secondly there were strong theoretical grounds for believing in the ways in which the statistical equating process was working. The result was therefore that ‘masterly inactivity’, taking a longer-term view of the issue, and collecting more data in order to understand better, was the appropriate strategy.

Nearly five years after statistical equating was introduced to Part 1 it seems that it can be stated that the changes in the pass rate for non-UK candidates are not an artefactual, non-intended consequence of the change in the standard-setting process. The differences shown by equating are probably real for Part 1. That a similar, albeit smaller, change occurred also in non-UK candidates taking Part 2 is probably explained best by the fact that pass rates for Part 2 were also rising fairly consistently over the time period looked at.

Examination processes thrive on stability where little changes either in the examinations themselves or the candidates taking them. Under such conditions the processes can be seen to be working well. In a complex social world where many changes are happening to candidates for many reasons, it would hardly be surprising if changes in pass rates occurred because changes occurred in candidates. When such changes coincide with a procedural change in an assessment then it is tempting to attribute the pass rate change to the procedural change, although that is only one of a range of hypotheses. If theory is strong enough, so that the procedural change can be trusted with confidence, then it is unwise to assume the pass rate change is because of the procedural change. The human mind is subject to a wide range of ‘cognitive illusions’, and seeing patterns in graphs with small numbers of data points is one of them, particularly when there is a large arrow pointing to the time when some other change had been introduced. Although we were concerned about possible changes for a number of years, the net conclusion is probably that the changes were only a cognitive illusion, one which we can now stand back and see for what it truly was.

### What does this analysis say about the Angoff/Hofstee process?

Although Angoff and Hofstee are both well-recognised methods in extensive use for standard-setting, that does not necessarily mean that they are either valid or justifiable in practice. Recent studies [16], including one which used MRCP(UK) data [17], have suggested that in the absence of normative data the judgements of item difficulty in the Angoff process are close to being random, but that if normative, performance data are presented then “judges may be displaying an over-reliance on the [performance] data, essentially replacing their content-based judgments with norm-referenced judgements” [41] (p.33. Kane [27] has argued that standard-setting methods, as with any other educational methods, require validation. That however rarely occurs, and most validation of judgemental methods such as Angoff rely for their validation mainly on repeated assertion of validity of process rather than any formal demonstration. The present study allowed a direct comparison of the predictive validity for the MRCP(UK) of a standard set by its hybrid Angoff/Hofstee method with a standard set by statistical equating. Predictive validity for the latter was higher than for the former, providing formal evidence of the benefits to the MRCP(UK) of introducing statistical equating.

### Limitations of the present study

An obvious limitation of the present study is that it is confined to two examinations at a single postgraduate institution, albeit one that is very large and its examinations are held internationally. Whether that substantially reduces the generalizability of our conclusions will become apparent as further institutions publish their research. We are also aware that although we have looked at predictive validity in terms of earlier assessments predicting outcomes of later ones, we have not studied the subsequent clinical and professional behaviour of the candidates who pass the exams. That is not easy to do, although there are two interesting examples in the literature [6, 42] where examination results have been predictive of professional behaviours and patient outcome. We would hope to be able to collect such data for MRCP(UK) at some time in the future, although inevitably there is a long time lag involved.

A different form of limitation is that we have not attempted to assess whether the shifts in performance of non-UK graduates are related to changes in demographic or educational measures in this pool of candidates. The MRCP(UK) inevitably has relatively little data on these candidates, since MRCP(UK) is acting as an examination board and not a training programme. More problematic is that candidates self-select, for a host of little understood reasons, to take MRCP(UK) (and the examination is open to anyone who feels they have adequate English, and has a recognised primary medical qualification). Anything which alters that self-selection might alter the standard of candidates, but in the absence of denominators it is nearly impossible to analyse. Likewise, non-UK candidates have qualified at a very large number of different medical schools around the world, and there is little quality data on the overall standard of graduates from those medical schools, making it difficult to interpret any shifts in the mix of candidates.

A reviewer raised the question of whether the Angoff process in our exams was ‘sub-optimal’, having only a small number of judges, with a median of 7 for Part 1 and 10 for Part 2. Several recent reviews have not mentioned the appropriate number of judges [2, 15, 43], and another review [44] cites earlier studies saying that acceptable numbers are “5 to 10”, “no less than 5 and no more than 30”, “20 to 25” and “as many judges as resources permit” before itself concluding that “should use at least 10 and ideally 15 to 20 judges” (p.68). A recent paper by Clauser et al. [45] described 18 Angoff panels at the NBME, which had a median number of judges of 9 (range 7 to 11). We are not convinced that our application of Angoff was atypical of many uses of the procedure. It may well be sub-optimal, with Clauser et al. [45] suggesting that there is substantial variation *between panels*, with their D-study considering 3 panels of 30 judges to reduce error substantially, but on that basis almost every Angoff process would be found wanting.

## Conclusions

The introduction of statistical equating to the MRCP(UK) written examinations was relatively straightforward and has resulted in a method of standard-setting which has a better theoretical underpinning, results in greater predictive validity than did the previous Angoff/Hofstee method, requires less examiner time, and reveals subtleties about the examination process, such as variation in pass rates within the annual academic cycle, that previously were less clear. The co-occurrence of an apparent step-change in pass rate for non-UK candidates immediately after equating was introduced, in both Part 1 and Part 2 exams, did result in some concerns, but a detailed analysis over a longer time-scale suggests that the changes were not related to statistical equating itself but reflected longer-term changes in true ability of the candidates.

## Abbreviations

ABIM: American Board of Internal Medicine
DIF: Differential item functioning
ETS: Educational testing service
IRT: Item response theory
MRCP(UK): Membership of the royal colleges of physicians of the United Kingdom
NBME: National board of medical examiners
OSCE: Objective structured clinical examination
PACES: Practical assessment of clinical and examination skills
UK: United Kingdom
UKCAT: United Kingdom clinical aptitude test.
